# Unique profile on the progress free survival and overall survival in patients with advanced non-small cell lung cancer in the Qujing area, Southwest China

**DOI:** 10.3389/fimmu.2023.1012166

**Published:** 2023-02-28

**Authors:** Yuhui Ma, Hutao Shi, Guangqiang Zhao, Xin Liu, Jingjing Cai, Guangjian Li, Wanlin Chen, Yujie Lei, Lianhua Ye, Chaojiang Fu, Li Zhao, Yongchun Zhou, Yunchao Huang

**Affiliations:** ^1^ Department of Thoracic Surgery I, The Yunnan Cancer Hospital, Kunming, China; ^2^ Department of Imaging at Kunming Tongren Hospital, Kunming, China; ^3^ Yunnan Cancer Hospital and The Third Affiliated Hospital of Kunming Medical University, Yunnan Cancer Center, Kunming, China; ^4^ Emergency Department (Outpatient Chemotherapy Center) at Yunnan Cancer Hospital, Kunming, China; ^5^ Department of Anesthesiology at Yunnan Cancer Hospital, Kunming, China

**Keywords:** immune signature, predictive biomarker, NSCLC, immunotherapy, Qujing

## Abstract

**Background:**

China’s southwestern region, Qujing, harbors a high incidence of non-small cell lung cancer (NSCLC) and related mortality. This study was designed to reveal the impact of an immune-related prognostic signature (IRPS) on advanced NSCLC in the Qujing.

**Methods:**

Tissue specimens from an independent cohort of 37 patients with advanced NSCLC were retrospectively evaluated to determine the relationship between the IRPS estimated by next-generation sequencing (NGS) and clinical outcome. To compare the IRPS in tissue and the clinical outcomes between Qujing and non-Qujing populations, we analyzed datasets of 23 patients with advanced NSCLC from The Cancer Genome Atlas (TCGA) database. In addition, an independent cohort (n=111) of blood specimens was retrospectively analyzed to determine the relationship between the IRPS and clinical outcome. Finally, we evaluated the utility of the blood IRPS in classifying 24 patients with advanced NSCLC who might benefit from immunotherapy.

**Results:**

In cohort 1, the Qujing population with tTMB-H (≥ 10 mutations/Mb) or KRAS mutations had shorter progression-free survival (PFS) (hazard ratio [HR] 0.37, 0.14 to 0.97, *P* = 0.04; HR 0.23, 0.08 to 0.66, *P* < 0.01) and overall survival (OS) (HR 0.05, 0.01 to 0.35, *P* < 0.01; HR 0.22, 0.07 to 0.66, *P* < 0.01). In cohort 2 of the Qujing population, bTMB-H (≥ 6 mutations per Mb) and KRAS mutations were related to PFS (HR 0.59, 0.36 to 0.99, *P* = 0.04; HR 0.50, 0.26 to 0.98, *P* = 0.04) and OS (HR 0.58, 0.35 to 0.96, *P* = 0.03; HR 0.48, 0.25 to 0.93, *P* = 0.03). Notably, the Qujing population with bTMB-H had superior PFS (HR 0.32, 0.09 to 1.09, *P* = 0.01), OS (HR 0.33, 0.10 to 1.13, *P* < 0.01) and objective response rates (ORRs) (83.3% vs. 14.3% vs. 20.0%, *P <*0.01) to immunotherapy than other populations.

**Conclusions:**

These findings show that tTMB, bTMB and KRAS mutations appear to be independent validated IRPSs that predict the clinical outcomes of Qujing populations with advanced NSCLC and that bTMB may be used as a reliable IRPS to predict the clinical benefit from anti-PD-1 therapies among populations from Qujing with advanced NSCLC.

## 1 Introduction

According to the most recent report published by the American Cancer Society (ACS) in 2021 and the Chinese annual cancer report in 2021 (incidence data up to 2018, mortality data up to 2019), lung cancer still has the second highest incidence rate and is the leading cause of cancer-related death worldwide (1). China’s southwestern region, including Qujing city, harbors an extremely high incidence of non-small cell lung cancer (NSCLC) and related mortality (2, 3). Previous real-world research showed that patients (n=2672) with NSCLC in the Qujing area have unique alterations in driver genes (4, 5). Patients with NSCLC in Qujing are more likely to carry the compound mutations EGFR G719X+S768I and EGFR G719X+L861Q in addition to 19DEL and L858R (4). Moreover, the proportion of KRAS (G12C) subtype mutations in NSCLC patients is higher in Qujing (4). Recently, immune checkpoint inhibitors (ICIs) have seen considerable success in treating advanced NSCLC, but unavoidable limitations still exist due to insufficient response rates (6). Some evidence indicates that alterations in some driver genes (TP53, KRAS, EGFR, SMAD4) in advanced NSCLC may impact the immune microenvironment and response to ICIs (7, 8). However, it is still unclear whether an immune-related prognostic signature (IRPS) in Qujing populations with advanced NSCLC is influenced by unique alterations in driver genes. Consequently, it is imperative that the IRPS of advanced NSCLC patients in the Qujing region be fully understood to determine the optimum immunotherapeutic regimen for these patients.

The IMpower110 study (n=572) found that a PD-L1 (programmed cell death ligand-1) inhibitor is effective as a first-line treatment for advanced NSCLC patients whose tumors have PD-L1 expression on ≥ 1% of tumor cells (9). Tissue tumor mutational burden (tTMB), in addition to PD-L1 expression, can also contribute to clinical benefits from multiple ICIs (10, 11). However, in clinical applications, existing biomarkers have limited value due to tumor heterogeneity, nonstandard cutoff levels, and unsatisfactory predictive power. Furthermore, a previous study also demonstrated that compared with NSCLC specimens from The Cancer Genome Atlas (TCGA), Qujing specimens exhibited a higher tTMB (median = 2.11) (12). Thus, this study was conducted to construct a risk model from a tissue IRPS (TMB, PD-L1, TP53, KRAS, EGFR and SMAD4) in Qujing patients with advanced NSCLC to establish a comprehensive prognostic biomarker that can predict ICI responsiveness in Qujing populations with advanced NSCLC.

Although recent clinical trials have shown a correlation between tTMB-High and improved clinical outcomes in advanced NSCLC patients receiving ICIs, up to 30% of advanced NSCLC patients are unlikely to have sufficient tissue specimens for biomarker analysis during diagnosis (13–15). It is therefore necessary that a noninvasive risk model be used for the Qujing population that can identify patients who would benefit from ICI treatment. Recently, based on the phase 2 B-F1RST (n=153) study, high blood TMB was related to improved clinical outcomes in advanced NSCLC patients receiving atezolizumab monotherapy (15). In this study, we established a noninvasive risk model using a blood IRPS (TMB, TP53, KRAS, EGFR and SMAD4) in Qujing patients with advanced NSCLC to better compensate for the shortfalls of existing prognostic signatures.

## 2 Methods

### 2.1 Study design

This study involved 2 cohorts. First, 37 patients (21 patients from the Qujing area) with advanced NSCLC were recruited, and tissue specimens were collected from March 2018 to June 2022 at the Yunnan Cancer Center for retrospective IRPS status analysis. From The Cancer Genome Atlas (TCGA) database, TCGA-advanced NSCLC (n=23) was analyzed for an IRPS comparison between the Qujing and Western populations (online **Supplement Tables S1**). Second, we recruited 111 advanced NSCLC patients with available blood specimens (45 patients from the Qujing area and 24 patients receiving ICI treatment) from the cancer center between March 2018 and June 2022 to retrospectively determine their blood IRPS status. Prior to the study, all patients (n=148) with advanced NSCLC received platinum-based chemotherapy as a first-line treatment. Informed consent was obtained from all patients, and the study was performed in full compliance with the Declaration of Helsinki. This protocol was approved by the Clinical Research Ethics Committee of Yunnan Cancer Center. The Yunnan patients with advanced NSCLC were considered eligible for the IRPS analysis if they had adequate tissue and blood samples. To obtain survival data, 37 tissue samples were analyzed for a tissue IRPS, and 111 blood samples were analyzed for a blood IRPS. Finally, we gathered other information on clinical and molecular factors, which is shown in the online **Supplement Tables S2-3**. Details of the methods of DNA extraction, library preparation, sequencing, data processing, immunohistochemistry, IRPS detection and outcome assessment are in the online **Supplement Methods S1-5**.

### 2.2 Statistical analysis

Statistical analyses were performed using GraphPad Prism (V.9.3.1) and R software (V.4.2.0). An analysis of the clinicopathological parameters and IRPS alterations was performed with the χ^2^ test. The two groups (TCGA-advanced NSCLC and Yunnan tissue-advanced NSCLC) were compared using a 2-tailed, unpaired t test for normal distribution or a Mann−Whitney test for nonnormal distribution. PFS and OS were evaluated using Kaplan−Meier curves, while prognostic and risk significance was assessed using the log-rank test and Cox regression model. In different subgroups based on the IRPS, the ORRs were compared using a χ^2^ test. Statistics were reported with 2-tailed *P* values, and *P* < 0.05 was considered statistically significant.

## 3 Results

### 3.1 Baseline characteristics

This study comprised two independent cohorts. There were 37 advanced NSCLC patients with tissue specimens in cohort 1; the median age was 58 (range 36-80) years, and 14 patients were female (37.8%). The second cohort included 111 advanced NSCLC patients with blood specimens; the median age was 56 (range 31-82) years, and 39 were female (35.1%). The following are details of the clinical factors; see online **Supplement Tables S2-3**.

### 3.2 Development of a tissue immune-related prognostic signature (tIRPS) in the Qujing population

**Figure 1:**
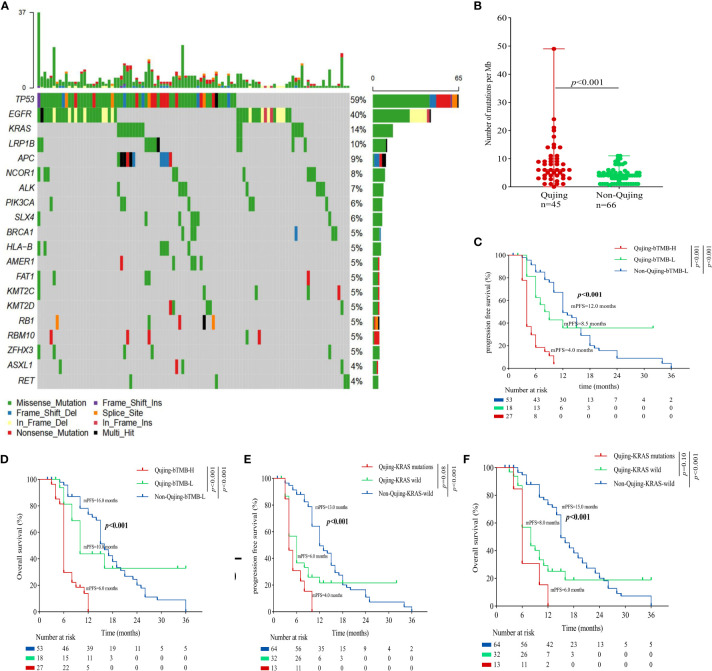
An overview of the molecular and survival characteristics in cohort 1 with advanced NSCLC. **(A)** Profile of comutations in cohort 1 cancer specimens. **(B)** Comparative analysis of tTMB between Qujing and non-Qujing populations. **(C)** A Kaplan−Meier analysis of PFS in cohort 1 by tTMB status. **(D)** A Kaplan−Meier analysis of OS in cohort 1 by tTMB status. **(E)** A Kaplan−Meier analysis of PFS in cohort 1 by KRAS status. **(F)** A Kaplan−Meier analysis of OS in cohort 1 by KRAS status.

To identify the Qujing tIRPS in advanced NSCLC patients who might experience a clinical benefit, the association between clinical and molecular characteristics in cohort 1 was first performed (**Figure 1A**; **online Supplement Table S4**). Then, the relationship between clinical and other known biomarkers (PD-L1 and TMB) in cohort 1 was tested. Compared with non-Qujing specimens, Qujing specimens displayed a higher TMB (median = 8.0, *P <* 0.001, **Figure 1B**). Additionally, based on prior studies and FDA guidelines (16, 17), the cutoff level for tTMB was 10 mutations/Mb in cohort 1. We found that Qujing populations were significantly more prone to tTMB-H (≥ 10 mutations/Mb) than non-Qujing populations in cohort 1 (*P* < 0.01, **online Supplement Table S4**).

Next, we performed a survival analysis of the candidate Qujing tIRPSs for PFS and OS in cohort 1. Compared with the other two populations, Qujing populations with tTMB-H exhibited a shorter PFS and OS (7.0 vs. 10.0 vs. 19.0 months, *P* < 0.001, 7.0 vs. 12.5 vs. 21.0 months, *P* < 0.001, **Figures 1C, D**). Moreover, compared with the other two populations, Qujing populations with KRAS mutations also exhibited shorter PFS and OS (4.0 vs. 9.0 vs. 20.0 months, *P* < 0.001, 4.0 vs. 11.0 vs. 28.0 months, *P* < 0.001, **Figures 1E, F**). Finally, we performed a Cox regression analysis of these candidate Qujing tIRPSs to determine PFS and OS in cohort 1. Interestingly, tTMB-H or KRAS mutations were independently correlated with PFS and OS in cohort 1 of the Qujing population (**Table 1**). Overall, our results suggested that tTMB-H and KRAS mutations could each serve as an independent tIRPS to predict clinical benefit among Qujing populations.

**Table 1:** Univariable and multivariable Cox regression analyses of the candidate Qujing IRPSs in cohort 1 for PFS and OS.

Variable	Univariable Analysis		Multivariable Analysis	
	HR (95%CI)	*P* value	HR (95%CI)	*P* value
Progression-free survival				
Qujing vs. non-Qujing	4.88 (1.63 to 14.57)	<0.01^a^	4.36 (1.29 to 14.76)	0.01^a^
EGFR (mutation vs. wild type)	0.53 (0.21 to 1.31)	0.17	NA	NA
KRAS (mutation vs. wild type)	2.57 (1.00 to 6.64)	0.05^a^	0.23 (0.08 to 0.66)	<0.01^a^
SMAD4 (mutation vs. wild type)	2.04 (0.58 to 7.15)	0.26	NA	NA
TP53 (mutation vs. wild type)	1.82 (0.75 to 4.43)	0.18	NA	NA
TMB-H vs. TMB-L	3.83 (1.61 to 9.09)	<0.01^a^	0.37 (0.14 to 0.97)	0.04^a^
PD-L1 (≥ 1% vs. < 1%)	0.68 (0.22 to 2.08)	0.50	NA	NA
Overall survival				
Qujing vs. non-Qujing	6.20 (2.06 to 18.68)	<0.01^a^	9.49 (2.32 to 38.72)	<0.01^a^
EGFR (mutation vs. wild type)	0.60 (0.25 to 1.47)	0.27	NA	NA
KRAS (mutation vs. wild type)	2.50 (0.97 to 6.44)	0.05^a^	0.22 (0.07 to 0.66)	<0.01^a^
SMAD4 (mutation vs. wild type)	1.96 (0.56 to 6.79)	0.28	NA	NA
TP53 (mutation vs. wild type)	1.94 (0.80 to 4.72)	0.14	NA	NA
TMB-H vs. TMB-L	19.98 (4.28 to 93.16)	<0.01^a^	0.05 (0.01 to 0.35)	<0.01^a^
PD-L1 (≥ 1% vs. < 1%)	0.58 (0.18 to 1.83)	0.36	NA	NA

Variables with a significance level of P ≤ 0.05 in the univariable analysis were entered into the multivariable analysis. PD-L1, Programmed cell death ligand 1. tTMB-H, Tissue tumor mutational burden-high (cutoff value ≥ 10 mutations/Mb). NA, not applicable. ^a^P value indicates a statistically significant difference.

### 3.3 Validation of the predictive value of the tIRPS for the Qujing population

**Figure 2:**
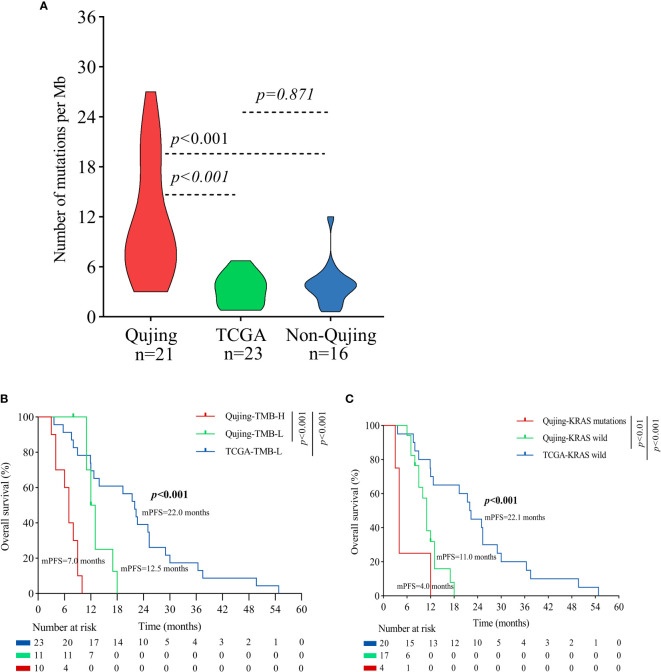
An overview of the molecular and survival characteristics between cohort 1 and the TCGA cohort with advanced NSCLC. **(A)** Violin plot displaying the tTMB of the Qujing, TCGA and non-Qujing populations. **(B)** A Kaplan−Meier analysis of OS between the Qujing and TCGA populations by tTMB. **(C)** A Kaplan−Meier analysis of OS between the Qujing and TCGA populations by KRAS status.

Moreover, to validate the predictive value of the tIRPS in relation to a clinical benefit in the Qujing population, we compared the difference in the tIRPS between the TCGA cohort and cohort 1-Qujing. A significant number of tIRPS characteristics (including TMB-H and PD-L1 < 1%) occurred in Qujing populations compared with the Western cohort (**online Supplement Table S5**). Furthermore, in comparison with TCGA specimens, Qujing specimens displayed a higher TMB (median = 8.0, *P* < 0.001, **Figure 2A**). Next, using survival curves, we analyzed the OS of cohort 1 and the TCGA cohort based on these candidate tIRPSs. Compared with Western populations, Qujing populations with tTMB-H exhibited a shorter OS (7.0 vs. 12.5 vs. 22.0 months, *P* < 0.001, **Figure 2B**). Moreover, compared with Western populations, Qujing populations with KRAS mutations also exhibited a shorter OS (4.0 vs. 11.0 vs. 28.0 months, *P* < 0.001, **Figure 2C**). Finally, we performed a Cox regression analysis of these candidate Qujing tIRPSs for OS in cohort 1 and the TCGA cohort. Interestingly, compared with the TCGA cohort and non-Qujing populations, tTMB-H or KRAS mutations were independently correlated with OS in Qujing populations (**Table 2**). Overall, the results of this study validate that tTMB-H and KRAS mutations have the potential to serve as independent tIRPSs to predict a clinical benefit among the Qujing population.

**Table 2:** Univariable and multivariable Cox regression analyses of candidate Qujing IRPSs in two cohorts (n=60) for OS.

Variable	Univariable Analysis	*P* value	Multivariable Analysis	*P* value
	HR (95%CI)		HR (95%CI)	
**Overall survival**				
Qujing vs. TCGA+non-Qujing	5.24 (2.47 to 11.12)	<0.001^a^	0.27 (0.11 to 0.64)	0.003^a^
EGFR (mutation vs. wild type)	1.29 (0.67 to 2.45)	0.43	NA	NA
KRAS (mutation vs. wild type)	2.52 (1.10 to 5.74)	0.02^a^	0.36 (0.15 to 0.84)	0.019^a^
SMAD4 (mutation vs. wild type)	2.56 (0.76 to 8.60)	0.12	NA	NA
TP53 (mutation vs. wild type)	1.46 (0.80 to 2.64)	0.20	NA	NA
TMB-H vs. TMB-L	10.83 (4.05 to 28.94)	<0.001^a^	6.18 (2.13 to 17.88)	0.001^a^
PD-L1 (≥ 1% vs. < 1%)	0.38 (0.14 to 1.01)	0.054	NA	NA

Variables with a significance level of P ≤ 0.05 in the univariable analysis were entered into the multivariable analysis. PD-L1, Programmed cell death ligand 1. tTMB-H, Tissue tumor mutational burden-high (cutoff value ≥ 10 mutations/Mb). NA, not applicable.^a^P value indicates a statistically significant difference.

### 3.4 Development of a blood immune-related prognostic signature (bIRPS) in Qujing populations

**Figure 3:**
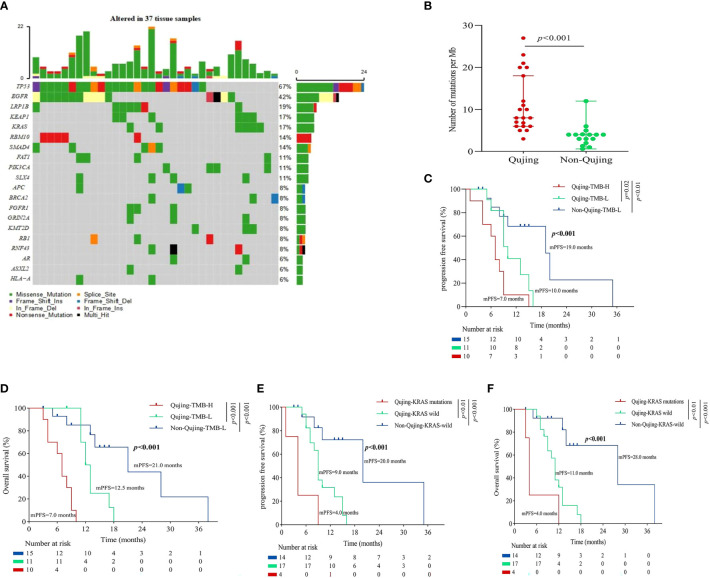
An overview of the molecular and survival characteristics in patients with advanced NSCLC in cohort 2. **(A)** Profile of comutations in blood specimens from cohort 2. **(B)** Comparative analysis of bTMB between Qujing and non-Qujing populations. **(C)** A Kaplan−Meier analysis of PFS in cohort 2 by bTMB status. **(D)** A Kaplan−Meier analysis of OS in cohort 2 by bTMB status. **(E)** A Kaplan−Meier analysis of PFS in cohort 2 by KRAS status. **(F)** A Kaplan−Meier analysis of OS in cohort 2 by KRAS status.

To identify a blood IRPS in advanced NSCLC patients in Qujing who would experience a clinical benefit, a test was first performed for the association between clinical and molecular characteristics. We found that Qujing populations were significantly more prone to KRAS mutations than non-Qujing populations (*P* < 0.001, **Figure 3A**; **online Supplement Table S6**). Then, the relationship between clinical and other known biomarkers (blood TMB) in cohort 2 was tested. Compared with non-Qujing specimens, Qujing specimens displayed a higher bTMB (median = 6.0, *P* < 0.001, **Figure 3B**). Additionally, based on prior studies (14, 18), the cutoff level for bTMB was 6 mutations/Mb in cohort 2. We found that Qujing populations were significantly more prone to bTMB-H (≥ 6 mutations/Mb) than non-Qujing populations in cohort 2 (*P <* 0.001, **online Supplement Table S6**).

Next, we performed a survival analysis of these candidate Qujing bIRPSs for PFS and OS in cohort 2. Compared with the other two populations, Qujing populations with bTMB-H displayed a shorter PFS and OS (4.0 vs. 8.5 vs. 12.0 months, *P* < 0.001, 6.0 vs. 10.0 vs. 16.0 months, *P* < 0.001, **Figures 3C, D**). Moreover, compared with the other two populations, Qujing populations with KRAS mutations also showed a shorter PFS and OS (4.0 vs. 6.0 vs. 12.0 months, *P* < 0.001, 6.0 vs. 8.0 vs. 15.0 months, *P* < 0.001, **Figures 3E, F**). Finally, we performed a Cox regression analysis of these candidate Qujing bIRPSs for PFS and OS in cohort 2. Interestingly, in Qujing populations, bTMB-H and KRAS mutations were independently correlated with PFS and OS in cohort 2 (**Table 3**). Overall, the results of these findings validate that bTMB-H and KRAS mutations have the potential to serve as independent bIRPS to predict clinical benefit among the Qujing population.

**Table 3:** Univariable and multivariable Cox regression analyses of candidate bIRPSs for PFS and OS in the Qujing populations in cohort 2.

Variable	Univariable Analysis	*P* value	Multivariable Analysis	*P* value
	HR (95%CI)		HR (95%CI)	
Progression-free survival				
Qujing vs. non-Qujing	0.45 (0.29 to 0.70)	<0.01^a^	1.83 (1.11 to 3.00)	0.01^a^
EGFR (mutation vs. wild type)	0.98 (0.64 to 1.50)	0.94	NA	NA
KRAS (mutation vs. wild type)	0.29 (0.16 to 0.53)	<0.01^a^	0.50 (0.26 to 0.98)	0.04^a^
SMAD4 (mutation vs. wild type)	NA	NA	NA	NA
TP53 (mutation vs. wild type)	0.53 (0.34 to 0.81)	<0.01^a^	0.66 (0.40 to 1.07)	0.09
bTMB ≥6 vs. <6	0.41 (0.26 to 0.63)	<0.01^a^	0.59 (0.36 to 0.99)	0.04^a^
Overall survival				
Qujing vs. non-Qujing	2.19 (1.41 to 3.39)	<0.01^a^	1.86 (1.13 to 3.07)	0.01^a^
EGFR (mutation vs. wild type)	1.03 (0.67 to 1.57)	0.87	NA	NA
KRAS (mutation vs. wild type)	0.28 (0.15 to 0.52)	<0.01^a^	0.48 (0.25 to 0.93)	0.03^a^
SMAD4 (mutation vs. wild type)	NA	NA	NA	NA
TP53 (mutation vs. wild type)	0.50 (0.32 to 0.77)	0.02^a^	0.62 (0.38 to 1.01)	0.06
bTMB ≥ 6 vs. <6	0.39 (0.25 to 0.60)	<0.01^a^	0.58 (0.35 to 0.96)	0.03^a^

Variables with a significance level of P ≤ 0.05 in the univariable analysis were entered into the multivariable analysis. bTMB-H, blood tumor mutational burden-high (cutoff value ≥ 6 mutations/Mb). NA, not applicable.

^a^P value indicates a statistically significant difference.

### 3.5 Validation of the predictive value of the blood immune-related prognostic signature (bIRPS) for ICI treatment

**Figure 4:**
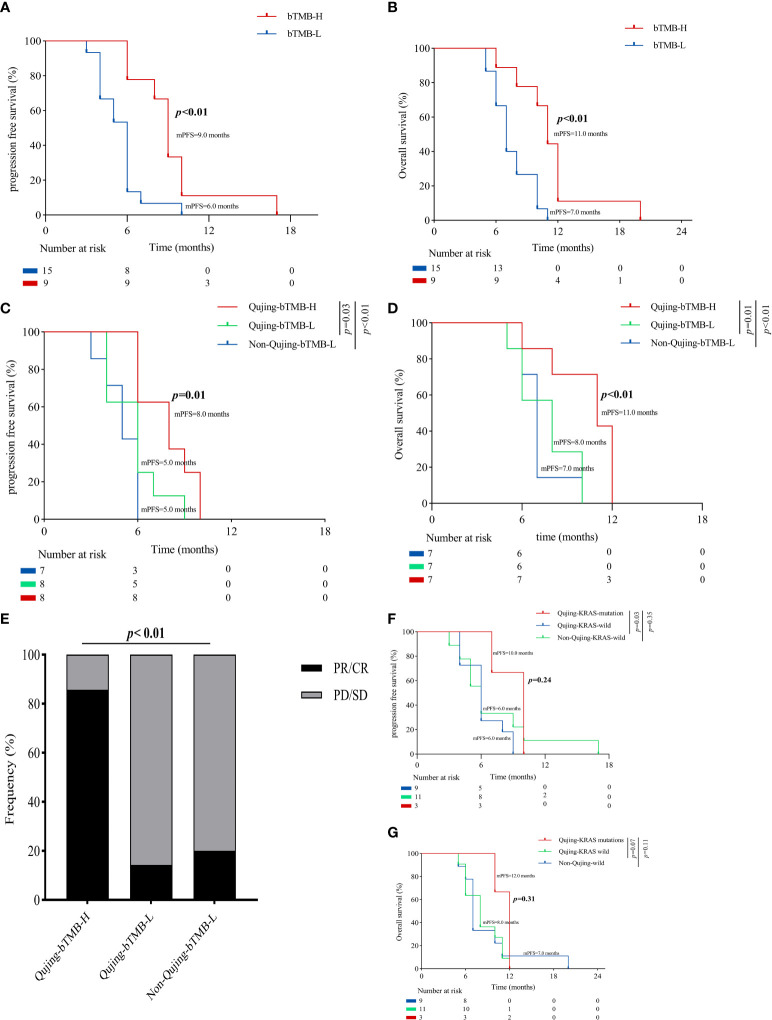
An overview of the survival characteristics in populations with advanced NSCLC treated with ICIs. **(A)** A Kaplan−Meier analysis of PFS in populations with advanced NSCLC by bTMB status. **(B)** A Kaplan−Meier analysis of OS in populations with advanced NSCLC by bTMB status. **(C)** A Kaplan−Meier analysis of PFS between Qujing and non-Qujing populations by bTMB status. **(D)** A Kaplan−Meier analysis of OS between Qujing and non-Qujing populations by bTMB status. **(E)** Comparison of ORRs between Qujing and non-Qujing populations. **(F)** A Kaplan−Meier analysis of PFS between Qujing and non-Qujing populations by KRAS status. **(G)** A Kaplan−Meier analysis of OS between Qujing and non-Qujing populations by KRAS status.

To demonstrate that the Qujing bIRPS can predict which patients with advanced NSCLC could benefit clinically from ICI therapy, a survival analysis for PFS and OS was first performed. Compared with the bTMB-L populations, advanced NSCLC patients exhibited a longer PFS and OS (9.0 vs. 5.5 months, *P* < 0.001, 11.0 vs. 7.0 months, *P* < 0.01, **Figures 4A, B**). Compared with the other two populations, Qujing populations with bTMB-H exhibited a longer PFS and OS (8.0 vs. 6.0 vs. 5.0 months, *P* = 0.01, 11.0 vs. 8.0 vs. 7.0 months, *P* < 0.01, **Figures 4C, D**). A comparison of ORRs also showed that Qujing populations in the bTMB-H subgroup achieved a higher proportion of partial response/complete response (PR/CR) than the other two groups (83.3% vs. 14.3% vs. 20.0%, *P <* 0.01, **Figure 4E**). Although Qujing populations with KRAS mutations also had a longer PFS and OS than the other two populations, the difference was not statistically significant (10.0 vs. 6.0 vs. 6.0 months, *P* = 0.24, 12.0 vs. 8.0 vs. 7.0 months, *P* = 0.31, **Figures 4F, G**). These findings suggested that bTMB might have greater predictive value for ICI treatment in Qujing populations.

## 4 Discussion

Even though immunotherapy, especially ICIs, represents a breakthrough in the treatment of NSCLC, only a fraction of patients will attain long-term benefits from immunotherapy. It is noteworthy that three distinctive characteristics of NSCLC are observed in the Qujing area, Yunnan: a higher incidence, a higher mortality, and a similar incidence for men and women. Nevertheless, in the absence of a retrospective study, the IRPS status of NSCLC has not yet been comprehensively explored among this area’s populations. Therefore, it is imperative to investigate a more precise IRPS in Qujing populations.

A previous study found that populations in Qujing with advanced NSCLC had a distinct mutational spectrum (higher EGFR G719X+S768I, EGFR G719X+L861Q and KRAS) compared with patients in other parts of Yunnan Province. In this multicohort retrospective analysis, we first established and validated a new Qujing IRPS model (tTMB, bTMB and KRAS mutations) that is capable of accurately predicting clinical outcomes among Qujing populations and stratifying those who might benefit from ICIs. Notably, rather than using whole-genome assays, the Qujing IRPS may significantly reduce the costs by simplifying the panel to two biomarkers, which can be used in clinical practice at a broad scale and at an affordable price.

Recent molecular evidence has demonstrated that cancer driver genes can regulate the tumor immune environment. For example, KRAS mutations promote inflammation and inhibit the immune response, eventually leading to cancer progression (19). However, one alteration may not be enough to induce a difference and is unlikely to be a precise prognostic predictor of ICI efficacy. Therefore, the model we developed includes most of the possible crucial IRPSs, and we have identified two IRPSs (TMB and KRAS mutations) that can accurately predict the clinical outcomes of patients with advanced NSCLC from Qujing and effectively differentiate ICI-sensitive patients. To our knowledge, for the first time, we discovered that the tTMB (cutoff level ≥ 10 mutations per Mb) is a powerful and independent predictive tIRPS in cohort 1 of Qujing advanced NSCLC patients not treated with ICIs. Moreover, compared with non-Qujing populations and TCGA populations, Qujing populations displayed a higher tTMB (median = 8.0 vs. 4.0 vs. 3.2, *P* < 0.001). While these findings are consistent with previous research (12), the median tTMB value (8.0 vs. 2.1) was significantly higher in our study. Differences in baseline characteristics may explain the differences in median tTMB values between our findings (IIIB-IV) and those of previous studies (female and I-IV). In addition, to our knowledge, we discovered for the first time that KRAS mutations constitute a powerful and independent predictive tIRPS in Qujing populations with advanced NSCLC not treated with ICIs. Based on these findings, evidence indicates that the unique alterations in KRAS in Qujing populations with advanced NSCLC may be associated with the immune microenvironment and clinical outcomes. Further investigation is ongoing to learn why Qujing populations with KRAS mutations have worse outcomes than other non-Qujing populations.

Several recent studies have shown that 30% to 50% of advanced NSCLC populations lack adequate cancer tissue for tIRPS detection (18, 20). Therefore, researchers have devoted considerable attention to investigating whether a bIRPS is an effective biomarker that can predict clinical benefit. To our knowledge, we discovered for the first time that both bTMB (cutoff level ≥ 6 mutations per Mb) and KRAS mutations were powerful independent bIRPSs that can predict outcomes in Qujing populations with advanced NSCLC from cohort 2 not treated with ICIs. Moreover, compared with non-Qujing populations, Qujing populations displayed a higher bTMB (median = 6.0 vs. 4.0, *P* < 0.001). In cohort 2, even though the bIRPS was validated for predicting survival in the Qujing population, both PFS and OS were different from those in cohort 1. In contrast to cohort 1, cohort 2 had a shorter survival time (PFS and OS). Since IRPSs are significantly less sensitive in blood samples than in tissue samples, when IRPS alterations are detected in blood samples, this indicates advanced cancer stage and decreased survival time. Differences in sample type may explain the differences in survival time between the two cohorts. Despite the lower sensitivity of detecting an IRPS in blood compared with tissue, a bIRPS is still an effective supplement to tIRPS due to its noninvasive, real-time and repeatable characteristics and can be widely adopted in clinical practice.

The cutoff points for bTMB are still debatable. According to Wang et al. (18), advanced NSCLC patients with bTMB-H (≥ 6 mutations per Mb) could benefit from ICIs. Subsequently, the phase 2 B-F1RST trial reported that advanced NSCLC patients with bTMB-H (≥ 16 mutations per Mb) could benefit from ICIs (15). Based on our study, 6 mutations per Mb was the cutoff point for bTMB, which is an appropriate value for distinguishing Qujing populations with advanced NSCLC from those in other regions in Yunnan who may benefit from ICIs. It is likely that two factors account for the difference between our study and the B-FIRST trial in regard to the bTMB cutoff threshold. First, the races of the patients included in our study (all populations were Chinese) and the B-FIRST trial (most populations were White) were different, which may have biased the selection of the bTMB cutoff threshold. Second, patients with EGFR mutations and ALK fusions, which have been shown to be associated with low TMB and low PD-L1 expression, were not excluded from this study (21).

To our knowledge, in the ICI therapy section, we discovered for the first time that bTMB was a powerful independent bIRPS that predicts clinical benefit (including PFS, OS and ORR) in advanced NSCLC patients in Qujing populations treated with ICIs. Despite different populations and cutoff points, the findings (clinical benefit) in this study are consistent with the results of the B-FIRST study. Nevertheless, despite that Qujing populations with KRAS mutations had a longer PFS and OS, a statistically significant difference was not observed. Our study suggests that two factors account for this limitation. First, the small sample size of this study (24 patients who received ICIs) might not sufficiently reflect whether ICIs are effective in Qujing populations with KRAS mutations. Second, this study was a retrospective single-center study, which may have contributed to the statistical discrepancy. Although KRAS mutations were not validated as an effective IRPS in Qujing advanced NSCLC populations treated with ICIs, this has opened up new possibilities for developing an ICI-TKI cotreatment modality. It was shown in an immunocompetent mouse model that the KRAS inhibitor AMG510 led to a more inflamed tumor immune environment, which resulted in a greater efficacy of ICIs (22). Multicenter prospective large-scale cotreatment research is expected.

Our real-world research has several major limitations, including its retrospective single-center design, small sample size and lack of blood PD-L1 expression data. Firstly, the limited sample size of cohort 1 may lead to unavoidable selection bias, resulting in relatively weakening the reliability of our conclusions. A limited sample size is currently available for cohort 1 due to the fact that up to 30% of advanced NSCLC patients lack sufficient second biopsy specimens for biomarker analysis. Therefore, the TCGA-advanced NSCLC database were used in this study for comparison in an effort to reduce the impact of data selection bias. Secondly, technical limitations prevented us from analyzing blood PD-L1 biomarkers. Hence, we do not know whether blood PD-L1 is an efficacy biomarker for ICI monotherapy after chemotherapy. Finally, the lack of blood sample data in the public database precluded us from comparing and verifying the cohort 2 research results. Therefore, further investigation based on multicenter, large-scale, independent, prospective studies is ongoing.

## 5 Conclusions

For the first time, tTMB, bTMB and KRAS mutations appear to be independent validated IRPSs of the clinical outcome for Qujing advanced NSCLC populations, and bTMB may be used as a reliable IRPS that predicts the clinical benefit from ICIs among Qujing populations with advanced NSCLC.

## Data availability statement

The datasets presented in this article are not readily available because of Chinese national law. Requests to access the datasets should be directed to corresponding author: YH, e-mail: hycyn2008@163.com

## Ethics statement

The studies involving human participants were reviewed and approved by Clinical Research Ethics Committee of Yunnan Cancer Center. The patients/participants provided their written informed consent to participate in this study.

## Author contributions

Study was designed by YH, YZ, HS, GZ, and YM. Experimental work was completed by YM, XL, JC, GL, WC, YL, LY, CF, and LZ. YM analyzed the parameters and wrote the paper. All authors contributed to the research. All authors contributed to the article and approved the submitted version.
