# Supplementary material for: Unique profile on the progress free survival and overall survival in patients with advanced non-small cell lung cancer in the Qujing area, Southwest China
Source: Front Immunol. 2023 Feb 28;14:1012166. doi: 10.3389/fimmu.2023.1012166 (PMC10011462; doi:10.3389/fimmu.2023.1012166)
Supplement: Supplementary file 1 [file DataSheet_1.doc]

**Supplementary Online Content**

**eMethods 1.** DNA extraction and NGS library construction

**eMethods 2.** Sequencing and data processing

**eMethods 3.** TMB caculation

**eMethods 4.** PD-L1 Immunohistochemical Analysis

**eMethods 5.** Outcomes Assessment

**eTable S1.** clinical data for TCGA-advanced NSCLC

**eTable S2.** Demographic and clinicopathologic characteristics of 37 advanced NSCLC in two cohorts

**eTable S3.** Detailed Clinicopathologic Features of 18 advanced NSCLC Patients with ICBs

**eTable S4.** Alteration frequency of Qujing tissue IRPS in cohort 1

**eTable S5.** Alteration frequency of tissue IRPS in two cohorts

**eTable S6.** Alteration frequency of Qujing blood IRPS in cohort 2

**eMethods 1. DNA Extraction and NGS library construction**

Following the manufacturer’s instructions, the QIAampDNA FFPE Tissue Kit was used to obtain genomic DNA from formalin-fixed paraffin-embedded (FFPE) tissue samples. The FFPE tissue sections were cut into 5μm thickness, and more than 20% of the cells were tumor cells. Detection of DNA concentrations (>300ng and 10 ng/μL) was carried out by the Qubit dsDNA HS Assay Kit. Input of DNA ranged from 50 to 200ng. Target enrichment was performed on DNA input < 50ng with an additional PCR cycle. Following that, TIANSeq DirectFast DNA Library Prep Kit was used to prepare enzyme-sheared DNA and sequencing libraries. Quantification of nucleic acid was performed with VAHTS library Quantification Kits for Illumina when the Ct level of the library was less than 10.

ctDNA extraction and purification from plasma (Thermo (k0782)), enrichment of whole blood leukocyte, and DNA was extracted and quality identified by low-speed centrifugation (Qubit dsDNA HS Assay Kit) before library construction. Vazyme ND607 DNA Library Kit was used to prepare ctDNA libraries containing unique identifiers (UIDs, also called barcodes) that identified each DNA molecules. A KAPA enzyme was used to digest the extracted DNA for poly(A) tail addition and end repair. PCR enrichment amplification was conducted after hybridization of target fragments captured with magnetic beads.

**eMethods 2. Sequencing and data processing**

The IRPS was evaluated with capture probes supplied by Integrated DNA Technologies (IDT) xGen Lockdown Probes (including 547 cancer-relevant genes), following standard protocols, tissue gDNA and plasma ctDNA were captured separately using the probes. Following manufacturer instructions, both plasma ctDNA and tissue gDNA libraries were processed into the Illumina® HiSeq X-TEN for sequencing. With Illumina® HiSeq X-TEN, raw parameters were processed, and error reads were corrected with hg19 reference gnome. These data were analyzed with KEYseq V2.0 in this study.

**eMethods 3.** TMB caculation

In the TMB calculation, synonymous and nonsynonymous variants with a frequency of > 5% (0.5% in blood) were summarised, as shown by mutations per megabase (mut/Mb). The bTMB algorithm filters common fake mutations based on single nucleotide polymorphisms (SNPs) and driver gene mutations, while synonymous mutations, small fragments and single base insertion-deletion mutations were not removed. The bTMB algorithm is built using official standards from Foundation Medicine, a leading provider of genome sequencing analysis services.

**eMethods 4.** PD-L1 Immunohistochemical Analysis

The Ventana SP263 assay was used on the BenchMark platform (Ventana, Tucson, AZ, USA) to evaluate the expression of PD-L1. The PD-L1 expression positivity means over 1% of tumor cells. For PD-L1 immunohistochemical analysis, samples must contain at least 100 viable cancer cells evaluated by two experienced pathologists. To evaluate the expression of PD-L1, 50 patients’ samples were available.

**eMethods 5. Outcomes Assessment**

Using the Response Evaluation Criteria In Solid Tumors (RECIST) v1.1, the PFS of patients without ICI therapy was calculated as the interval from IRPS detection to death or progressive disease. OS was determined from the time of detection of IRPS to the time of death for any reason. ICI-treated patients’ PFS was calculated as the period between the start of ICI therapy and objective death or progressive disease. ICI-treated patients’ OS was calculated as the period of time from the start of ICI treatment to death caused by any reason. ORR measured by investigator assessment is the percentage of patients who achieving a complete response (CR) or a partial response (PR) based on RECIST v1.1. Following IRPS detection, tumor assessments were conducted every 3 months, and the last follow-up occurred on June 16, 2022.

**eTable S2. Demographic and clinicopathologic characteristics of 148 advanced NSCLC patients in two cohorts.**

| | **Total** | **Cohort 1** | **Cohort 2** |  | | --- | --- | --- | --- | | **Characteristic** |  | **(tissue)** | **(blood)** | |
| --- | --- | --- | --- | --- | --- | --- | --- | --- |
| | **Characteristic** |  |  |  | | --- | --- | --- | --- | | No. of patients, N (%) | 148 | 37 (25.0%) | 111 (75.0%) | | **Age at initiation of IRPS test**: Median (range), y | 56 (31-82) | 58 (36-80) | 56 (31-82) | | **Sex, N (%)** |  |  |  | | Male | 95 (64.2%) | 23 (24.2%) | 72 (75.8%) | | Female | 53 (35.8%) | 14 (26.4%) | 39 (73.6%) | | **Histology, N (%)** |  |  |  | | Adenocarcinoma | 116 (78.4%) | 28 (24.1%) | 88 (75.9%) | | Squamous cell carcinoma | 26 (17.6%) | 8 (30.8%) | 18 (69.2%) | | NSCLC (NOS) | 6 (4.0%) | 1 (16.7%) | 5 (83.3%) | | **Smoking history, N (%)** |  |  |  | | Never | 75 (50.7%) | 16 (21.3) | 59 (78.7%) | | Former/current | 73 (49.3%) | 21 (28.8%) | 52 (71.2%) | | **TMB (mutations/Mb), median (range)** | 5 (0-49) | 6 (0.6-27) | 4 (0-49) | | **PD-L1 expression** |  |  |  | | > 50% | 6 (4.1%) | 2 (33.3%) | 4 (66.7%) | | 1%-49% | 20 (13.5%) | 11 (55.0%） | 9 (45.0%) | | < 1% | 23 (15.5%) | 8 (34.8%) | 15 (65.2%) | | Unknown | 99 (66.9%) | 16 (16.2%) | 83 (83.8%) | | **ECOG performance status, N (%)** |  |  |  | | 0-1 | 135 (91.2%) | 33 (24.4%) | 102 (75.6%) | | 2-4 | 13 (8.8%) | 4 (30.8%) | 9 (69.2%) | |

PD-L1, Programmed cell death ligand 1. TMB, tumor mutational burden. NSCLC, non–small-cell lung

cancer; NOS, nototherwise specified.

**eTable S3. Detailed Clinicopathologic Features of 24 advanced NSCLC Patients with ICBs.**

| | **Total** |  | | --- | --- | | **Characteristic** |  | |
| --- | --- | --- | --- | --- |
| | No. of patients, N (%) | 24 | | --- | --- | | **Age at initiation of IRPS test**: Median (range), y | 56 (31-79) | | **Sex, N (%)** |  | | Male | 17 (70.8%) | | Female | 7 (29.2%) | | **Histology, N (%)** |  | | Adenocarcinoma | 15 (62.5%) | | Squamous cell carcinoma | 8 (33.3%) | | NSCLC (NOS) | 1 (4.2%) | | **Smoking history, N (%)** |  | | Never | 13 (54.2%) | | Former/current | 11 (45.8%) | | **Immunotherapy (anti-PD-1 antibodies)** |  | | Nivolumab | 5 (20.8%) | | Pembrolizumab | 19 (79.2%) | | **TMB (mutations/Mb), median (range)** | 5.0 (1-20) | |

TMB, tumor mutational burden. NSCLC, non–small-cell lung cancer; NOS, nototherwise specified.

**eTable S4. Alteration frequency of Qujing tissue IRPS in cohort 1.**

| | **Total** | **Qujing** | **Non-Qujing** | **Chi-Squqre** |  | | --- | --- | --- | --- | --- | |  |  |  |  | ***P* value** | |
| --- | --- | --- | --- | --- | --- | --- | --- | --- | --- | --- |
| | No. of patients, N (%) | 37 | 21 (56.8%) | 16 (43.2%) |  | | --- | --- | --- | --- | --- | | **TP53** |  |  |  |  | | Wild type | 13 (35.1%) | 5 (38.5%) | 8 (61.5%) | 0.164 | | Mutation | 24 (64.9%) | 16 (66.7%) | 8 (33.3%) | | **EGFR** |  |  |  |  | | Wild type | 22 (59.5%) | 14 (63.6%) | 8 (36.4%) | 0.335 | | Mutation | 15 (40.5%) | 7 (46.7%) | 8 (53.3%) | | **KRAS** |  |  |  |  | | Wild type | 31 (83.8%) | 17 (54.8%) | 14 (45.2%) | 0.679 | | Mutation | 6 (16.2%) | 4 (66.7%) | 2 (33.3%) | | **SMAD4** |  |  |  |  | | Wild type | 33 (89.2%) | 18 (54.5%) | 15 (45.5%) | 0.618 | | Mutation | 4 (10.8%) | 3 (75.0%） | 1 (25.0%) | | **TMB** |  |  |  |  | | tTMB > 10 mutations/Mb | 11 (29.7%) | 10 (90.9%) | 1 (9.1%) | <0.01^a^ | | tTMB < 10 mutations/Mb | 26 (70.3%) | 11 (42.3%) | 15 (57.7%) | | **PD-L1 expression** |  |  |  |  | | PD-L1 > 1% | 13 (35.1%) | 5 (38.5%) | 8 (61.5%) | 0.06 | | PD-L1 < 1% | 8 (21.6%) | 7 (87.5%) | 1 (12.5%) | | unknown | 16 (43.2%) | 9 (56.3%) | 7 (43.7%) |  | |

^a^ This *p* value indicates a statistically significant difference. PD-L1, Programmed cell death ligand 1. tTMB, Tissue tumor mutational burden.

**eTable S5. Alteration frequency of tissue IRPS in two cohorts.**

| | **Total** | **Qujing** | **TCGA** | **Chi-Squqre** |  | | --- | --- | --- | --- | --- | |  |  |  |  | ***P* value** | |
| --- | --- | --- | --- | --- | --- | --- | --- | --- | --- | --- |
| | No. of patients, N (%) | 44 | 21 (47.7%) | 23 (52.3%) |  | | --- | --- | --- | --- | --- | | **TP53** |  |  |  |  | | Wild type | 16 (36.4%) | 5 (31.3%) | 11 (68.7%) | 0.098 | | Mutation | 28 (63.6%) | 16 (57.1%) | 12 (42.9%) | | **EGFR** |  |  |  |  | | Wild type | 34 (77.3%) | 14 (41.2%) | 20 (58.8%) | 0.155 | | Mutation | 10 (22.7%) | 7 (70.0%) | 3 (30.0%) | | **KRAS** |  |  |  |  | | Wild type | 37 (84.1%) | 17 (45.9%) | 20 (54.1%) | 0.692 | | Mutation | 7 (15.9%) | 4 (57.1%) | 3 (42.9%) | | **SMAD4** |  |  |  |  | | Wild type | 41 (93.2%) | 18 (43.9%) | 23 (56.1%) | 0.100 | | Mutation | 3 (6.8%) | 3 (100.0%） | 0 (0.0%) | | **TMB** |  |  |  |  | | tTMB > 10 mutations/Mb | 10 (22.7%) | 10 (100.0%) | 0 (0.0%) | <0.001^a^ | | tTMB < 10 mutations/Mb | 34 (77.3%) | 11 (32.4%) | 23 (67.6%) | | **PD-L1 expression** |  |  |  |  | | PD-L1 > 1% | 27 (61.4%) | 5 (18.5%) | 22 (81.5%) | <0.001^a^ | | PD-L1 < 1% | 7 (15.9%) | 7 (100.0%) | 0 (0.0%) | | unknown | 10 (22.7%) | 9 (90.0%) | 1 (10.0%) |  | |

^a^ This *p* value indicates a statistically significant difference. PD-L1, Programmed cell death ligand 1. tTMB, Tissue tumor mutational burden.

**eTable S6. Alteration frequency of Qujing blood IRPS in cohort 2.**

| | **Total** | **Qujing** | **Non-Qujing** | **Chi-Squqre** |  | | --- | --- | --- | --- | --- | |  |  |  |  | ***P* value** | |
| --- | --- | --- | --- | --- | --- | --- | --- | --- | --- | --- |
| | No. of patients, N (%) | 111 | 45 (40.5%) | 66 (59.5%) |  | | --- | --- | --- | --- | --- | | **TP53** |  |  |  |  | | Wild type | 44 (39.6%) | 15 (34.1%) | 29 (65.9%) | 0.262 | | Mutation | 67 (60.4%) | 30 (44.8%) | 37 (55.2%) | | **EGFR** |  |  |  |  | | Wild type | 68 (61.3%) | 28 (.41.2%) | 40 (58.8%) | 0.863 | | Mutation | 43 (38.7%) | 17 (39.5%) | 26 (60.5%) | | **KRAS** |  |  |  |  | | Wild type | 96 (86.5%) | 32 (33.3%) | 64 (66.7%) | <0.001^a^ | | Mutation | 15 (13.5%) | 13 (86.7%) | 2 (13.3%) | | **SMAD4** |  |  |  |  | | Wild type | 111 (100.0%) | 45 (40.5%) | 66 (59.5%) | 1.00 | | Mutation | 0 (0.0%) | 0 (0.0%） | 0 (0.0%) | | **Blood TMB** |  |  |  |  | | bTMB > 6 mutations/Mb | 40 (36.0%) | 27 (67.5%) | 13 (32.5%) | <0.001^a^ | | bTMB < 6 mutations/Mb | 71 (64.0%) | 18 (25.4%) | 53 (74.6%) | |

^a^ This *p* value indicates a statistically significant difference. bTMB, Blood tumor mutational burden.
